# Influence of Weight-Bearing Status After Tension Band Wiring of Patellar Fractures in Elderly Patients

**DOI:** 10.7759/cureus.94090

**Published:** 2025-10-08

**Authors:** Ahmed Zainy, Hannah Sadik, Luca Doltu, Chi Hoi Lee, Mohammed Al-Azzawi, Aroon Baskaradas

**Affiliations:** 1 Trauma and Orthopedics, London North West University Healthcare NHS Trust, London, GBR; 2 Trauma and Orthopedics, Imperial College London, London, GBR; 3 Trauma and Orthopedics, Stepping Hill Hospital, Manchester, GBR; 4 Trauma and Orthopedics, Manchester University NHS Foundation Trust, Manchester, GBR; 5 Trauma and Orthopedics, Royal Surrey County Hospital, Guildford, GBR

**Keywords:** early weight bearing, elderly patients​, fracture healing factors, functional outcomes, patella fracture, tension band wire

## Abstract

Background

Patellar fractures can severely affect mobility in the elderly, particularly due to compromised bone quality. Tension band wiring (TBW) is commonly used to repair displaced transverse fractures and permits early mobilization; however, the optimal timing for weight-bearing in elderly patients has not been clearly established. This study evaluated the outcomes of immediate versus delayed weight-bearing following TBW.

Methods

A retrospective cohort study was conducted involving elderly patients (≥60 years) from two hospitals in London. Patients were categorized into immediate (Day 0 post-op, n = 14) and delayed (>Day 0 post-op, n = 10) weight-bearing groups. Primary outcomes included knee range of motion (ROM), degree of extensor lag, reoperation rate, and time to union. Secondary outcomes included loss of fixation, implant failure, time to initiate knee flexion, and adherence to the rehabilitation protocol.

Results

Overall, patients in the immediate weight-bearing (IWB) group achieved union faster (8.4 weeks versus 13.7 weeks; p = 0.01), experienced less loss of reduction (n = 0, 0% versus n = 3, 30%; p = 0.03), had greater knee ROM (120.5° versus 101.3°; p = 0.02), and initiated knee flexion sooner (6.0 weeks versus 7.2 weeks; p < 0.001). There were no statistically significant differences between groups in extensor lag, need for reoperation, implant failure, or adherence to rehabilitation.

Conclusions

IWB after TBW of patellar fractures in elderly patients promotes fracture healing, knee function, and stability compared to traditional non-weight-bearing protocols and does not increase complication rates, supporting early mobilization strategies. Multicenter prospective studies with standardized fracture patterns are required to confirm these findings.

## Introduction

Patellar fractures represent approximately 1% of all fractures but can have significant consequences for functional mobility and severely impact quality of life, particularly in elderly patients who are likely to have poorer bone quality and diminished healing capacity [1]. The standard treatment for displaced transverse patellar fractures is tension band wiring (TBW), which converts tensile forces from the quadriceps into compressive forces at the fracture site, facilitating earlier mobilization of the knee joint [2]. However, the optimal timing of postoperative weight-bearing following TBW in elderly patients remains uncertain. Traditional weight-bearing protocols often involve prolonged immobilization, which has been shown to result in joint stiffness, muscle atrophy, and loss of functional baseline [3].

Most literature on postoperative rehabilitation for patellar fractures focuses on younger or mixed-age populations, providing limited evidence applicable to the elderly [4]. While some studies suggest that early mobilization can reduce complications, they do not specifically address outcomes in older patients [4,5]. Biomechanical studies indicate that early weight-bearing may decrease time to union and provide fracture stability without increasing complication rates; however, it is unclear whether these findings are applicable to elderly patients [6,7]. Conversely, delayed weight-bearing (DWB) has been associated with higher rates of fixation-related complications, often unintentionally, as fixation relies on normal loading to maintain stability [8].

The practical absence of evidence focused on elderly patients means that prescriptive guidance on postoperative rehabilitation strategies following TBW cannot be established. Therefore, this study aims to bridge this gap by comparing immediate weight-bearing (IWB) and DWB protocols in patients aged 60 years and older. Our hypothesis is that IWB will result in superior surgical and functional outcomes, based on biomechanical theory and preliminary evidence from recent fracture rehabilitation guidelines [8,9].

## Materials and methods

A retrospective observational cohort study was conducted at two district general hospitals under the same NHS Trust in London, UK, from January 1, 2022 to March 25, 2025. Data were obtained from electronic medical records. The clinical lead of the department granted approval for the study. Informed consent for the use of data was obtained at the time of surgery in accordance with World Medical Association policies. This study is reported in accordance with the STrengthening the Reporting of OBservational studies in Epidemiology (STROBE) recommendations.

Patient selection

Patients with the diagnosis code “Patella fracture” were initially screened, identifying a total of 458 cases. Patients aged ≥60 years (n = 214) were then selected. Further screening identified 28 patients who underwent TBW fixation. Four cases were excluded due to missing data, resulting in a final cohort of 24 patients. These were stratified into two groups based on the timing of weight-bearing initiation: immediate (weight-bearing starting on postoperative Day 0, n = 14, 58%) and delayed (weight-bearing started any time after Day 0, n = 10, 42%).

Exclusion criteria included age <60 years, conservative (nonoperative) management, surgical management with methods other than TBW, and missing outcome data.

Outcome measures

The primary outcomes were final knee range of motion (ROM), extensor lag, reoperation, and time to radiological union. Final knee ROM was measured in degrees from the final physiotherapy session notes and represents the maximum extension and flexion achieved. Extensor lag was measured similarly, with normal knee extension defined as 0°; any patient unable to fully straighten the knee was recorded as having extensor lag. Radiological union was documented and dated in clinic letters by senior orthopedic surgeons.

Secondary outcomes included loss of reduction, implant failure, time to initiation of knee flexion, and adherence to prescribed rehabilitation. Loss of reduction was identified on follow-up X-rays as displacement of the fracture. Implant failure included breakage or migration of K-wires and infection of metalwork. Time to initiation of knee flexion was defined as the week in which knee flexion exceeded 90°, recorded from physiotherapy notes. Adherence to rehabilitation was documented by physiotherapy staff; missed appointments or sessions were counted as noncompliance.

Statistical analysis 

Statistical analyses were performed using IBM SPSS Statistics for Windows, Version 30.0 (Released 2024; IBM Corp., Armonk, NY, USA). Continuous variables are reported as mean ± SD, and categorical variables as frequencies (percentages). Comparative analyses between the IWB and DWB groups were conducted using independent-samples t-tests for continuous variables and chi-square tests for categorical variables, as appropriate. Statistical significance was defined as a two-tailed p-value <0.05.

## Results

Baseline characteristics

A total of 24 elderly patients underwent TBW for patellar fractures. They were divided into the IWB (n = 14, 58%) and DWB (n = 10, 42%) groups. There were no significant differences in baseline demographics between the two groups (Table 1).

**Table 1:** Baseline patient characteristics DWB, delayed weight-bearing; IWB, immediate weight-bearing

Characteristics	IWB (n = 14)	DWB (n = 10)	p-Value
Age (years)	75.3 ± 6.5	77.8 ± 5.4	0.34
Female, n (%)	10 (71.4%)	8 (80%)	0.64
BMI (kg/m²)	24.8 ± 4.9	25.3 ± 4.1	0.78
Charlson Comorbidity Index	2.6 ± 1.2	2.9 ± 1.4	0.58

Surgical outcomes

Radiological union was achieved in all patients. IWB was associated with a significantly shorter mean time to union (8.4 ± 3.7 weeks, 95% CI: 6.3-10.5) compared to DWB (13.7 ± 4.9 weeks, 95% CI: 10.2-17.2; p = 0.01). IWB also reduced the incidence of loss of reduction (0%, n = 0, 95% CI: 0-23%) versus DWB (30%, n = 3, 95% CI: 7-65%; p = 0.03). There were no significant differences between groups in implant failure (IWB: n = 3, 21.4%, 95% CI: 5-51% vs. DWB: n = 3, 30%, 95% CI: 7-65%; p = 0.63) or reoperation rates (IWB: n = 4, 28.6%, 95% CI: 9-58% vs. DWB: n = 3, 30%, 95% CI: 7-65%; p = 0.97). The incidence of wound complications was low in both groups, with no statistical difference observed (Table 2).

**Table 2:** Surgical outcomes DWB, delayed weight-bearing; IWB, immediate weight-bearing

Outcomes	IWB (n = 14)	DWB (n = 10)	p-Value
Mean union time (weeks)	8.4 ± 3.7 (95% CI: 6.3-10.5)	13.7 ± 4.9 (95% CI: 10.2-17.2)	0.01
Loss of reduction, n (%)	0% (95% CI: 0-23%)	30% (95% CI: 7-65%)	0.03
Implant failure, n (%)	21.4% (95% CI: 5-51%)	30% (95% CI: 7-65%)	0.63
Reoperation, n (%)	28.6% (95% CI: 9-58%)	30% (95% CI: 7-65%)	0.97

Functional outcomes

Patients in the IWB group had significantly greater knee ROM at final follow-up (120.5 ± 15.8°, 95% CI: 111.4-129.6) compared to the DWB group (101.3 ± 16.2°, 95% CI: 89.7-112.9; p = 0.02) and initiated knee flexion earlier postoperatively (6.0 ± 0.0 weeks, 95% CI: 6.0-6.0 vs. 7.2 ± 1.4 weeks, 95% CI: 6.2-8.2; p < 0.001). Extensor lag did not differ significantly between groups (IWB: 14.3%, 95% CI: 1.8-42.8% vs. DWB: 20%, 95% CI: 2.5-55.6%; p = 0.71), nor did compliance with rehabilitation (IWB: 92.9%, 95% CI: 66.1-99.8% vs. DWB: 90%, 95% CI: 55.5-99.7%; p = 0.82) (Table 3).

**Table 3:** Functional outcomes DWB, delayed weight-bearing; IWB, immediate weight-bearing

Outcomes	IWB (n = 14)	DWB (n = 10)	p-Value
Knee range of movement	120.5 ± 15.8° (95% CI: 111.4-129.6)	101.3 ± 16.2° (95% CI: 89.7-112.9)	0.02
Initiation of knee flexion (weeks)	6.0 ± 0.0 (95% CI: 6.0-6.0)	7.2 ± 1.4 (95% CI: 6.2-8.2)	<0.001
Extensor lag, n (%)	2 (14.3%) (95% CI: 1.8-42.8%)	2 (20%) (95% CI: 2.5-55.6%)	0.71
Compliance with rehabilitation, n (%)	13 (92.9%) (95% CI: 66.1-99.8%)	9 (90%) (95% CI: 55.5-99.7%)	0.82

## Discussion

This study provides evidence supporting the safety and efficacy of immediate postoperative weight-bearing in elderly patients following TBW fixation of patellar fractures. Patients who began weight-bearing immediately demonstrated faster radiological union and greater knee ROM at final follow-up than those who remained non-weight-bearing, without a notable increase in adverse events. These findings are consistent with the principle that early mechanical loading stimulates bone healing and mitigates sequelae of prolonged immobilization [5,7,8].

Prior studies have indicated that early mobilization can prevent joint stiffness and muscle atrophy in the affected limb [1,3,8], and our results extend this understanding to an exclusively elderly cohort. There were no cases of fixation loss in the IWB group, whereas a few incidents occurred in the DWB group. This suggests that immediate postoperative weight-bearing does not compromise fixation stability in elderly patients, aligning with previous studies showing that stable fixation permits early mobilization without increasing hardware failure risk [5,8]. Evidence from other periarticular fractures similarly supports safe early weight-bearing when fixation is mechanically robust [7]. While the retrospective design precludes causal inference, the absence of fixation loss in the IWB group reinforces the interpretation that early loading, given stable constructs, is unlikely to jeopardize union.

Rehabilitation protocols for patellar fractures vary widely due to the paucity of robust data in elderly populations [4]. Nevertheless, there is a clear trend toward early mobilization, with modern guidelines recommending IWB, usually in full extension, and early knee exercises [8]. Our clinical data confirm that such an aggressive rehabilitation strategy is feasible and effective in older adults. Systematic reviews of lower limb fractures further indicate that early weight-bearing is associated with superior functional outcomes [7]. Our study demonstrates that IWB provides benefits in an older, high-risk population, paralleling outcomes observed in younger cohorts under similar protocols.

Limitations

This study has several limitations. First, the retrospective design and small sample size reduce the overall level of evidence and introduce potential selection bias. Second, patients were not randomized to IWB or DWB; rehabilitation decisions were made by treating surgeons and may have been influenced by intraoperative assessments of fracture stability. Consequently, the DWB group may have included more complex or comminuted fractures, potentially affecting outcomes. Third, follow-up focused on early healing and functional milestones without evaluating long-term outcomes such as chronic pain or late degenerative changes. These limitations indicate that, while encouraging, the findings should be interpreted with caution. Larger prospective, ideally randomized multicenter studies are needed to confirm the benefits of IWB and mitigate the influence of unmeasured confounders.

## Conclusions

IWB following TBW of patellar fractures in elderly patients improves fracture union time, stability, and functional outcomes without increasing complication rates. This study provides clinical evidence supporting early postoperative mobilization to maximize recovery and functional independence in this high-risk population. Further multicenter studies, ideally with standardized fracture patterns, are warranted to validate these findings and reduce potential bias.
